# Terlipressin relieves intestinal and renal injuries induced by acute mesenteric ischemia via PI3K/Akt pathway

**DOI:** 10.7150/ijms.46302

**Published:** 2020-09-28

**Authors:** Zi-Meng Liu, Han-Jin Lai, Xiang-Dong Guan, Shi-Hong Wen, Jian-Tong Shen, Yao Nie, Ning Liu, Xu-Yu Zhang

**Affiliations:** 1 Department of Critical Care Medicine, The First Affiliated Hospital, Sun Yat-sen University, No.58, Zhongshan 2nd Road, Guangzhou 510089, China.; 2 Department of Anesthesiology, The First Affiliated Hospital, Sun Yat-sen University, No.58, Zhongshan 2nd Road, Guangzhou 510089, China.

**Keywords:** vasopressor, V1 receptor, ischemia reperfusion injury, intestine, apoptosis, macrophage polarization

## Abstract

**Background:** To date, the effect of vasopressin on organ damages after acute mesenteric ischemia (MI) remains poorly understood.

**Aims:** To investigate the effect of terlipressin, a selective vasopressin V1 receptor agonist, versus norepinephrine on the intestinal and renal injuries after acute MI, and to explore the underlying mechanism of terlipressin.

**Methods:** Acute MI model was produced by clamping the superior mesenteric artery for 1 hour. Immediately after unclamping, terlipressin or norepinephrine was intravenously administered for 2 hours. Meanwhile, *in vitro*, RAW264.7 cells were treated with lipopolysaccharide or lipopolysaccharide+terlipressin. In addition, wortmannin was used to determine the role of phosphoinositide 3-kinase (PI3K)/ protein kinase B (Akt) pathway in the potential impacts of terlipressin.

**Results:** MI led to severe hypotension, caused notable intestinal and renal impairments and resulted in high mortality, which were markedly improved by terlipressin or norepinephrine. Terlipressin increased mean arterial pressure, decreased intestinal epithelial cell apoptosis, inhibited the generation of M1 macrophage in intestinal and renal tissues, and hindered the release of inflammatory cytokines after MI. Moreover, in cultured macrophages, terlipressin reduced the mRNA level of specific M1 markers and the release of inflammatory cytokines caused by lipopolysaccharide challenge. Wortmannin decreased the expression of PI3K and Akt induced by terlipressin in cells and in tissues, and abolished the above protective effects conferred by terlipressin.

**Conclusions:** Terlipressin or norepinephrine could effectively improve organ damages and mortality after acute MI. Terlipressin elevates blood pressure and inhibits intestinal epithelial apoptosis and macrophage M1 polarization via the PI3K/Akt pathway.

## Introduction

Acute mesenteric ischemia (MI), which can be subdivided into acute mesenteric arterial embolism and thrombosis, mesenteric venous thrombosis and nonocclusive mesenteric ischemia, according to the ischemic mechanism, is a life-threatening condition caused by impaired blood perfusion to the gut 1, 2. Acute MI and succeeding reperfusion may lead to vasodilatory hypotension, exacerbation of bacterial translocation and inflammatory responses, distant organ impairment and eventually multiple organ dysfunction 3, 4. As a consequence, acute MI is associated with high mortality for critically ill patients 1, 5. A retrospective study reported that 24.0% of decedents caused by sepsis in ICU were diagnosed with validated MI 6. Recently, another analysis showed that, in 214 ICU patients, acute MI was a secondary diagnosis in 58% of patients and the 30-day mortality rate was 71% 5. To maintain hemodynamic stability, vasopressors are commonly prescribed in the ICU. However, the use of some vasopressors may further decrease mesenteric flow and then aggravate the organ damages caused by MI 5, 7. Therefore, it is of clinical relevance to investigate the impacts of specific vasopressor on the injury of intestine and remote organs after the occurrence of acute MI.

In our recently published multicenter clinical study, we found that severe diarrhea was more common in the septic shock patients receiving terlipressin, a highly selective vasopressin V1 receptor agonist, than in those receiving traditional norepinephrine, although the suspected MI rates in two groups were similar 8. Consistent with our study, several case reports have demonstrated that V1 receptor agonists could cause severe bowel ischemic complications in the clinical settings 9-11. However, most of the experimental studies did not demonstrate any detrimental effect of vasopressin V1 agonist on splanchnic macro- and microcirculation 12. We previously found that terlipressin could protect intestinal epithelial cells against oxygen-glucose deprivation attack* in vitro* 13. Qiu* et al.* have demonstrated that terlipressin could promote intestinal microvascular flow in rats with endotoxic shock 14. So far, the definite effect of terlipressin on intestinal and other splanchnic injuries following acute MI insult has not been clarified. It is rather significant to investigate whether terlipressin increases MI-induced organ damages when compared with traditional vasopressor.

Therefore, we aimed to determine the effects of terlipressin on intestinal and renal injury with an acute MI model and to explore its underlying mechanisms. Norepinephrine was set as the control vasopressor.

## Materials and Methods

### Animal and cell line

The current animal experiments were approved by the Institutional Review Board of our university [2015A-059; President: X.Q. Yu] and was performed in accordance with National Institutes of Health guidelines for the experimental animal. Adult specific pathogen free (SPF) male Sprague-Dawley rats (300-350 g) were purchased and acclimated for 1 week before experiment entry.

The mouse RAW264.7 macrophage cell lines (ATCC, MD, USA) were maintained in Dulbecco's modified Eagle's medium containing fetal bovine serum, streptomycin and penicillin, and incubated at 37 °C in a humidified 5% CO_2_ incubator.

### Experimental Protocol

The present *in vivo* and *in vitro* experimental protocol was determined according to previous literatures and our pilot results.

The rats were anesthetized with intraperitoneal injection of 30 mg kg^-1^ pentobarbital and were mechanically ventilated with a standard ventilation protocol. Then, a catheter was inserted into the carotid artery and connected to a digital monitoring system for measuring hemodynamics, and the femoral vein was cannulated for drug infusion, as previously described 15. After midline laparotomy, the superior mesenteric artery (SMA) was identified. The rats were randomly allocated into 6 groups. Sham group (Sham): the animals underwent laparotomy without occlusion of the SMA, and received 0.5 mL normal saline (NS) injection followed by 0.75 mL h^-1^ continuous intravenous infusion for 2 hours. Acute MI group (MI): the SMA was clamped with a microaneurysm clip for 1 hour, and the animals received 0.5 mL normal saline (NS) immediately after unclamping of SMA followed by 0.75 mL h^-1^ for 2 hours. Terlipressin group (TP): Terlipressin (Hybio Pharmaceutical Co., Shenzhen, China) was dissolved in NS to 8 μg mL^-1^, and 0.5 mL terlipressin solution was given immediately after ischemia followed by 0.75 mL h^-1^ for 2 hours. Norepinephrine group (NE): Norepinephrine was dissolved in NS to 100 μg mL^-1^, and 0.5 mL norepinephrine solution was given immediately after ischemia followed by 0.75 mL h^-1^ for 2 hours. Wortmannin group (WT): 1.5 mg kg^-1^ phosphoinositide 3-kinase (PI3K) inhibitor wortmannin (MedChem Express, Shanghai, China) dissolved in 10% dimethyl sulfoxide (DMSO) 100 μL was administered intraperitoneally after unclamping, and then NS was administered. Telipressin+Wortmannin group (T+W): isometric wortmannin and terlipressin were administered after unclamping according to the above-mentioned regimen. At 2^nd^ hour after unclamping, the animals were euthanized by overdose of pentobarbital, and then the biological samples were harvested. No equivalent dose of terlipressin compared to norepinephrine has been reported. Thus, based on our preliminary hemodynamic data, the current dosing regimens of terlipressin and norepinephrine were designed to keep MAP within 15% of baseline level after ischemia. And each rat received equivalent volume of infusing solution (2 mL) during experimental period.

Confluent RAW264.7 cells were randomly assigned to 4 groups. Control group (Control): the cells received 0.1% DMSO and were incubated for 24 hours. Lipopolysaccharide group (LPS): the cells received 100 ng mL^-1^ LPS (E. coli O111:B4, Sigma Aldrich, MO, USA) and were incubated for 24 hours. Terlipressin group (Terlipressin): 50 nM terlipressin was administered to the medium immediately after LPS treatment, and then the cells were incubated for 24 hours. Telipressin+Wortmannin group (Telipressin+Wortmannin): 5 μM wortmannin was given at 1 hour before terlipressin treatment in the presence of LPS for 24 hours. After 24 hours incubation, the samples in each group were collected and detected.

### Histological analysis

Tissues were collected from a 0.5 cm ileum fragment (2 cm next to ileocecal valve) and upper pole of the left kidney. The segments were fixed in 4% paraformaldehyde and embedded in paraffin. The fixed tissues were sectioned and stained with hematoxylin-eosin. The histological damage was assessed by two independent and blinded pathologists using respective scoring system based on similar literatures (Table S1).

### Enzyme linked immunosorbent assay (ELISA) and biochemical analysis

Rat's tumor necrosis factor-α (TNF-α), interleukin (IL)-1β and IL-6 concentrations in rat's serum, the supernatants of tissue homogenates and culture medium were determined with commercial ELISA kits (R&D systems, MN, USA and Mlbio, Shanghai, China). 8-isoprostane, an accurate biomarker of lipid peroxidation, in rat's serum was detected with ELISA kit (ab175819, Abcam, Shanghai, China). The results were expressed as pg mL^-1^.

Rat's serum blood urea nitrogen (BUN) and creatinine levels were detected with an autoanalyzer (Hitachi, Tokyo, Japan). Diamine oxidase (DAO), an indicator of the integrity and functional mass of the intestine 16, in intestinal mucosal tissues was detected by a chemical assay kit (Jiancheng Bioengineering Institute, Nanjing, China). Results were expressed as U L^-1^.

### Western blot analysis

The proteins in the RAW264.7 cells, intestinal epithelia and kidney were detected by western blot. Briefly, the proteins were extracted from cells and tissues, and subjected to SDS-PAGE and electrophoretically transferred to nitrocellulose membranes. Membranes were blocked and incubated overnight with anti-PI3K antibody (4257, Cell Signaling Technology, MA, USA), anti-phospho-protein kinase B (p-Akt) antibody (13038, Cell Signaling Technology) or anti-cleaved caspase-3 antibody (9664, Cell Signaling Technology). After being washed three times, the membrane was incubated with a secondary antibody (7074, Cell Signaling Technology). Antibody dilutions used were according to user manual. Finally, the bands were imaged and quantified by Image J software (Ver 1.51, National Institutes of Health, MA, USA) and normalized with the glyceraldehyde-3-phosphate dehydrogenase (GAPDH).

### Quantitative polymerase chain reaction (qPCR)

The mRNA expression in RAW264.7 cells was detected through qPCR. In brief, total RNA was isolated by using TRIzol reagent (Invitrogen, CA, USA). Based on a similar study 17, quantitative PCR was performed by a C1000 Touch Thermal cycler (Bio-Rad Laboratories Inc, CA, USA) with SYBR Green Supermix to measure inducible nitric oxide synthase (iNOS), IL-1β, Arg1 and FIZZ1 mRNA levels. The relative expressions of mRNA transcripts were normalized by the internal control GAPDH.

### Immunohistochemistry and terminal deoxynucleotidyl transferase dUTP nick end labeling staining

Immunohistochemical examination was conducted to investigate the change of macrophages polarization 18. Briefly, the slices of intestine and kidney sections were incubated with anti-iNOS antibody (1:400, ab15323, Abcam, Shanghai, China) and CD163 antibody (1:400, ab182422, Abcam), followed by a secondary HRP-labeled antibody (1:200, ab205718, Abcam) for 1 hour at room temperature. Images were taken by using a microscope (BX51, Olympus Corporation, Tokyo, Japan) at a magnification of 400×. Finally, the number of positive cell (brown yellow) was counted by using Image-Pro Plus software. Quantification of cells expressing the specified marker was performed by calculating positive cells in five randomly chosen 400× fields of each sample.

The severity of apoptosis in the small intestine and kidney was respectively detected by terminal deoxynucleotidyl transferase biotin-dUTP nick end-labeling assay (TUNEL). The number of apoptotic cells was counted using Image-Pro Plus software (ver. 6.0, Media Cybernetics, MD, USA) and the apoptotic index was calculated accordingly (the number of apoptotic cell nuclei/the number of total cell nuclei ×100).

### Survival analysis

Briefly, the animals were treated with the above interventions and then transferred to the individual cage. Each rat was monitored via video recording for 24 hours 15.

### Statistical analysis

SPSS 18.0 software (SPSS Inc, IL, USA) was used for the data analysis. Hemodynamic data of rats was expressed as the mean ± standard error of the mean (SEM) and was analyzed by two-way ANOVA with repeated measures followed by Bonferroni posttest. Survival time from the beginning of unclamping were expressed as median (range), and results were compared by Kaplan Meier log-rank test. The survival rate was analyzed by Fisher exact test. The rest of the data such as biochemistry, histologic, western blot analysis and qPCR were expressed as mean ± SEM and analyzed by one-way ANOVA followed by Tukey posttest for multiple comparisons. *P*<0.05 was considered statistically significant.

## Results

### Terlipressin or norepinephrine improved hemodynamics after acute MI

**Figure 1:**
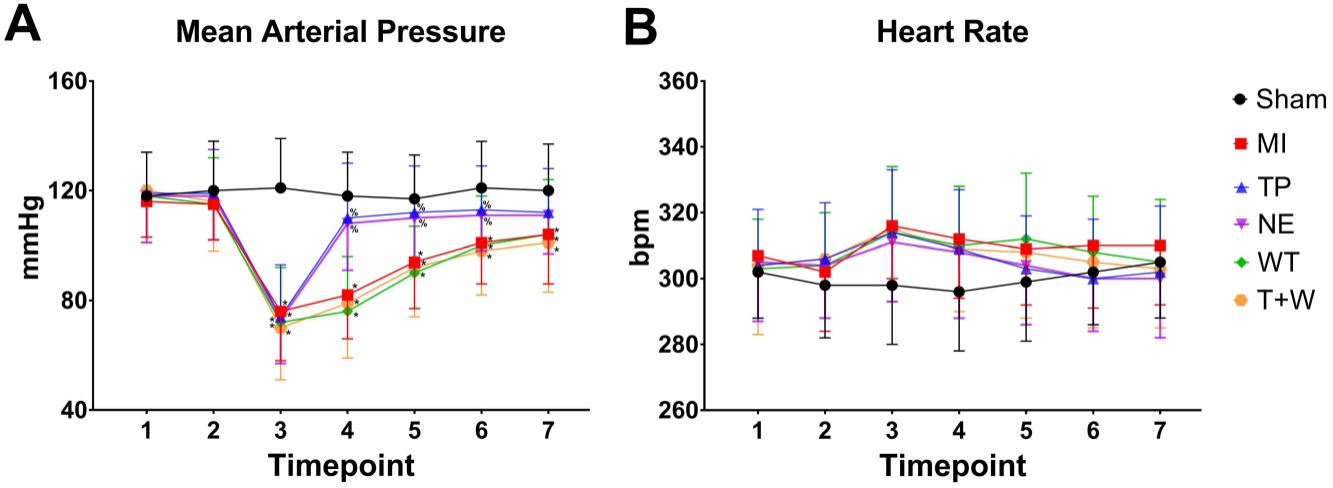
** Effects of terlipressin or norepinephrine infusion on mean arterial pressure (A) and heart rate (B) during the study period.** Animals underwent various interventions based on the experimental protocol. Data are expressed as mean±SEM, n=4 to 5. Results were compared by two-way ANOVA with repeated measures followed by Bonferroni posttest. * P<0.05 vs. Sham group; **%** *P<*0.05 vs. MI group, WT group or T+W group. Timepoint 1: baseline, 2: clamping the SMA, 3: immediately after unclamping the SMA, 4: 3 minutes after unclamping, 5: 30 minutes after unclamping, 6: 60 minutes after unclamping, 7: 120 minutes after unclamping. Sham group: the SMA of rat was exposed but not occluded; MI group: acute mesenteric ischemia model was produced by clamping the SMA; TP group: terlipressin was infused after unclamping the SMA; NE group: norepinephrine was infused after unclamping the SMA; WT group: wortmannin, a specific PI3K inhibitor, was used after mesenteric ischemia; T+W group: terlipressin and wortmannin were both administered after ischemia. SMA: superior mesenteric artery; PI3K: phosphoinositide 3-kinase.

As shown in Fig. 1, acute MI led to significantly decreased MAP (all *P*<0.05, MI vs. Sham; Fig. 1A) and slightly increased heart rate (all *P*>0.05; Fig.1B) throughout the whole reperfusion phase. The current dosage of norepinephrine or terlipressin effectively elevated MAP (all *P*<0.05, vs. MI; Fig. 1A), and there was no significant difference in MAP between TP group and NE group after MI (all *P*>0.05).

### Terlipressin or norepinephrine alleviated intestinal and renal injuries after acute MI

**Figure 2:**
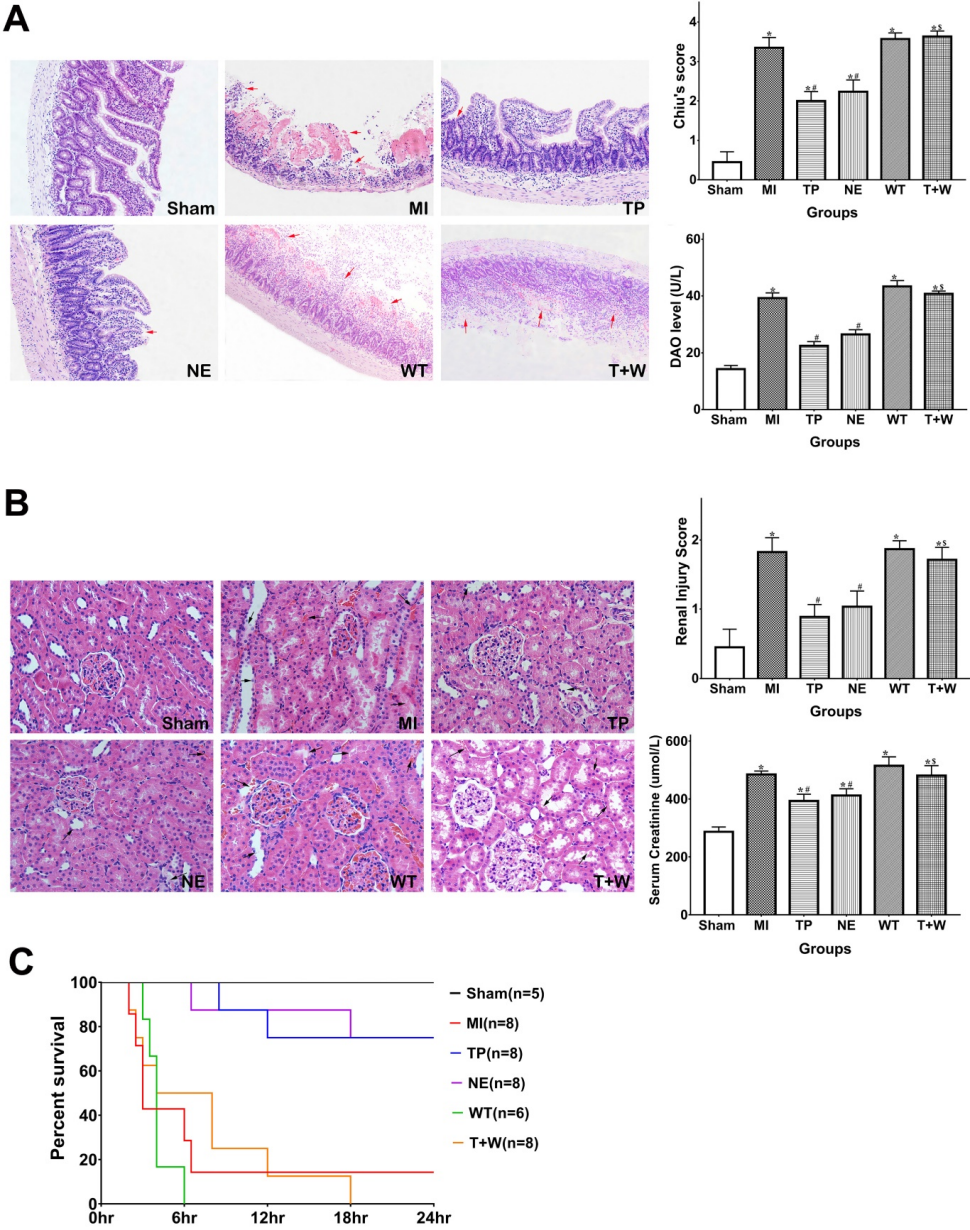
** Effects of terlipressin or norepinephrine infusion on intestinal and renal injuries, and mortality after acute MI.** (**A**) The ileal sections were stained with hematoxylin-eosin and evaluated with Chiu's scores under light microscopy (×100). In the upper panel, representative microscopic images from various groups were presented, and several morphologic damages of intestinal mucosa were indicated by red arrows. Analyses of Chiu's score and DAO level were presented in the lower panel. (**B**) The renal sections were also stained with hematoxylin-eosin and evaluated with renal injury scores under light microscopy (×200). In the upper panel, representative microscopic images from various groups were presented, and several morphologic damages of renal tissues were indicated by black arrows. Analyses of renal injury score and serum creatinine concentration were presented in the lower panel. Data were expressed as mean±SEM, n=4 to 8. Results were compared by ANOVA with Tukey posttest. * P < 0.05 vs. Sham group; # *P* < 0.05 vs. MI group; $ *P* < 0.05 vs. TP group. (**C**) Animals underwent different interventions based on the experimental protocol. Survival time is calculated from the initiation of reperfusion. Results were compared by Kaplan-Meier log rank test and Fisher exact test, n=5 to 8. Survival times in the TP and NE groups were significantly longer than those in the MI group, and the 24-h mortality rate was also decreased in the TP and NE groups. Whereas, the use of wortmannin diminished the improvement of survival delivered by terlipressin (T+W group). Sham group: the SMA of rat was exposed but not occluded; MI group: acute mesenteric ischemia model was produced by clamping the SMA; TP group: terlipressin was infused after unclamping the SMA; NE group: norepinephrine was infused after unclamping the SMA; WT group: wortmannin, a specific PI3K inhibitor, was used after mesenteric ischemia; T+W group: terlipressin and wortmannin were both administered after ischemia. MI: mesenteric ischemia; DAO: diamine oxidase; SMA: superior mesenteric artery; PI3K: phosphoinositide 3-kinase.

As shown in Figure 2A, the Chiu's score and DAO level in the MI group were significantly higher than that in the Sham group (both *P*<0.05). Terlipressin or norepinephrine significantly attenuated Chiu's score and DAO level (all *P*<0.05 vs. MI; Fig. 2A), and there was no statistical difference in Chiu's score and DAO level between group TP and NE (all *P*>0.05).

Meanwhile, the serum BUN and creatinine levels and the injury score of the kidney in the MI group were higher than that in the Sham group (all *P*<0.05; Fig. 2B). Either terlipressin or norepinephrine infusion significantly attenuated renal injuries (all P<0.05 vs. MI; Fig. 2B), and there was no statistical difference in these parameters between group TP and NE (all *P*>0.05; Fig. 2B).

The survival time and mortality rate of the rats in the MI group were 3 hours (2-24 hours) and 85.7%, respectively (Fig. 2C). Terlipressin or norepinephrine significantly increased survival time and survivors (both *P*<0.05 vs. MI; Fig. 2C).

### Terlipressin or norepinephrine reduced intestinal epithelial apoptosis after acute MI

**Figure 3:**
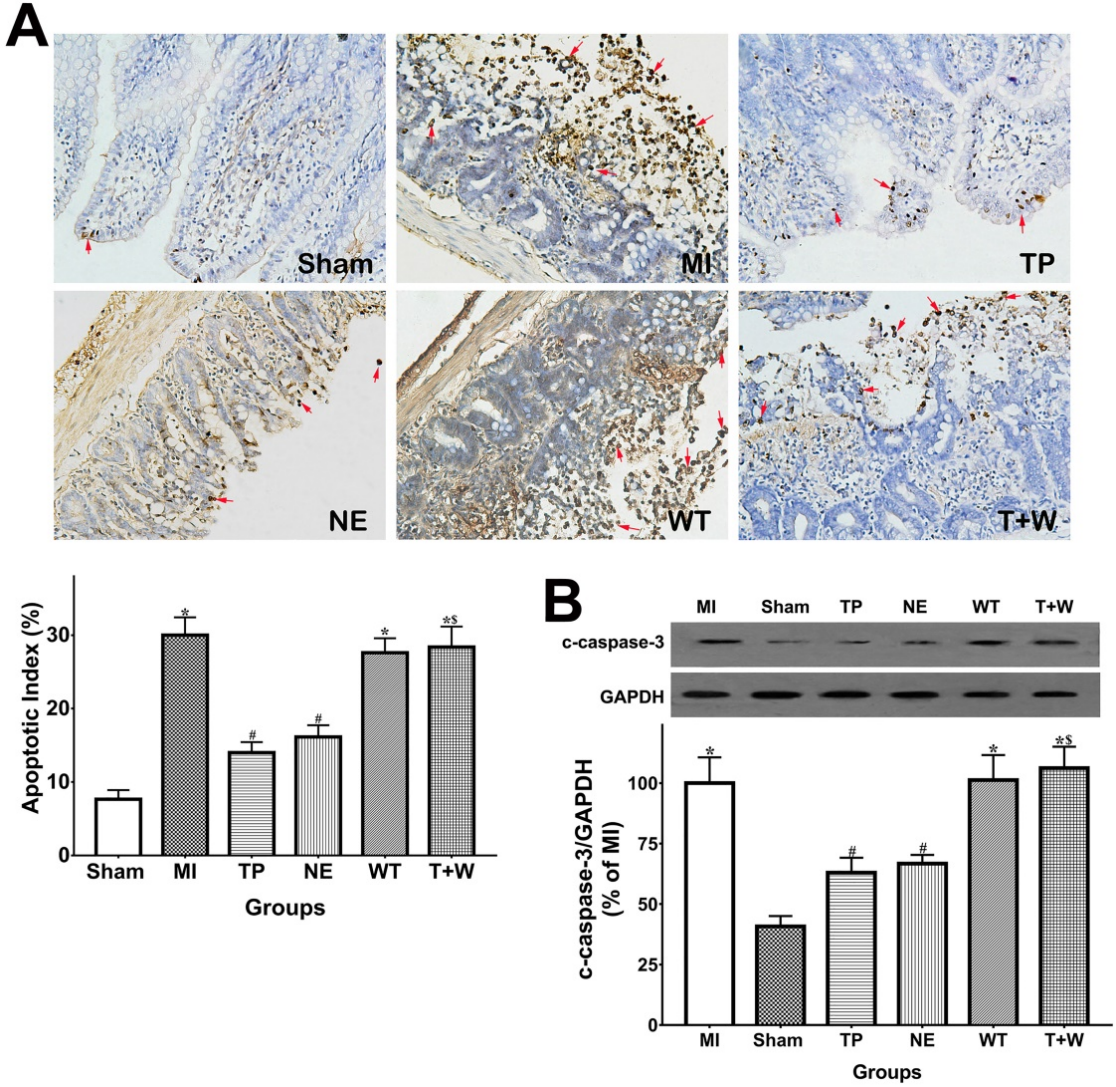
** Effects of terlipressin or norepinephrine infusion on intestinal epithelial apoptosis after acute MI.** (**A**) The ileal sections were stained by using TUNEL and evaluated with apoptotic epithelial cells under light microscopy (×200). Representative microscopic images from various groups were presented in the upper panel, and apoptotic nuclei were stained dark brown (red arrows). Analysis of apoptotic index was presented in the lower left-hand. (**B**) The cleaved caspase-3 expression in intestinal epithelial tissues was detected by western blotting assay. Representative bands of densitometry analysis from each group were shown in the upper side, and analysis of quantitative changes in caspase-3 expressions was shown in the bottom. Data were expressed as mean ± SEM, n=4 to 7. Results were compared by ANOVA with Tukey posttest. * *P* < 0.05 vs. Sham group; # *P* < 0.05 vs. MI group; $ *P* < 0.05 vs. TP group. Sham group: the SMA of rat was exposed but not occluded; MI group: acute mesenteric ischemia model was produced by clamping the SMA; TP group: terlipressin was infused after unclamping the SMA; NE group: norepinephrine was infused after unclamping the SMA; WT group: wortmannin, a specific PI3K inhibitor, was used after mesenteric ischemia; T+W group: terlipressin and wortmannin were both administered after ischemia. MI: mesenteric ischemia; SMA: superior mesenteric artery; PI3K: phosphoinositide 3-kinase; TUNEL: terminal deoxynucleotidyl transferase biotin-dUTP nick end-labeling; c-caspase-3: cleaved caspase-3; GAPDH: glyceraldehyde-3-phosphate dehydrogenase.

**Figure 4:**
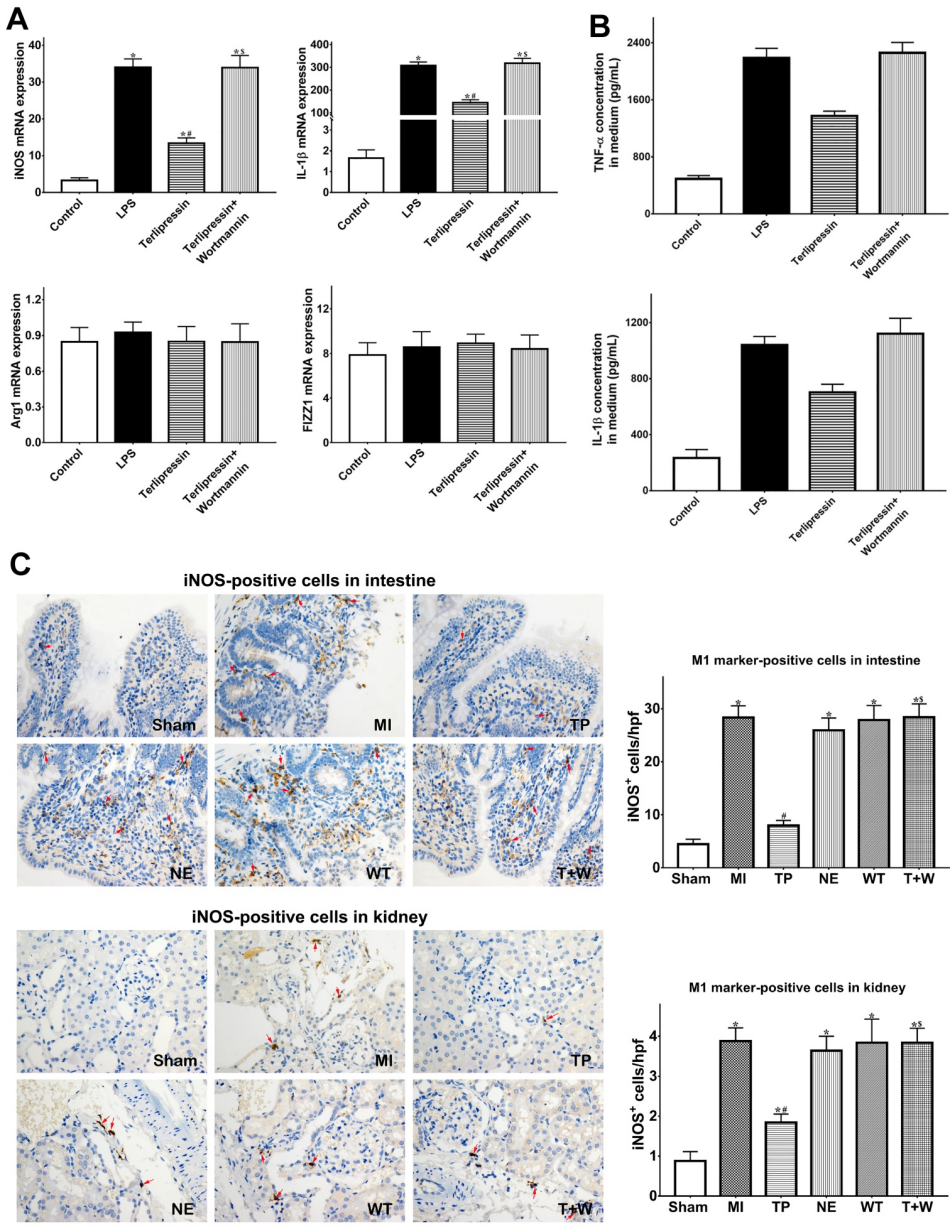

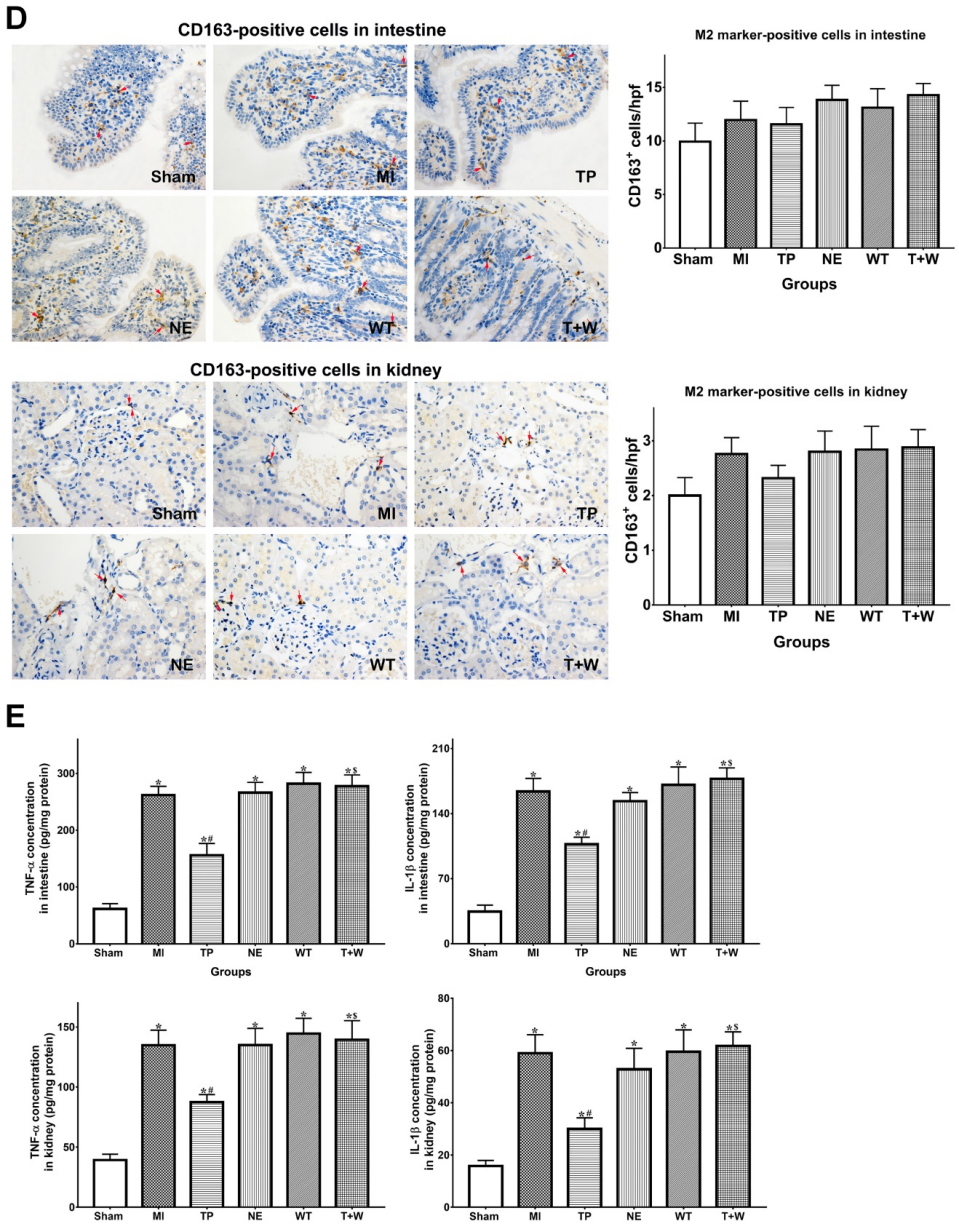
** Effects of terlipressin or norepinephrine on macrophage polarization in cultured cells after LPS attack and in organs after acute MI.** (**A**) The markers of M1 and M2 macrophages in RAW264.7 cells were detected by using PCR. Analyses of M1 markers (iNOS and IL-1β) and M2 markers (Arg1 and FIZZ1) mRNA expressions were presented. (**B**) The concentration of TNF-α and IL-1β in cultured medium was respectively detected by using ELISA assay. Analyses of TNF-α (upper) and IL-1β (lower) concentration in culture medium were presented. (**C**) Immunohistochemical stainings of iNOS was performed to detect iNOS-positive cells (M1 macrophages) in ileal and renal sections under light microscopy (×400). Representative microscopic images from various groups were presented and positive cells were stained brown-yellow (red arrows). Analyses of iNOS-positive cells in intestine and kidney were presented in the bottom. (**D**) Immunohistochemical staining of CD163 was performed to detect CD163-positive cells (M2 macrophages) in ileal and renal sections under light microscopy (×400). Representative microscopic images from various groups were presented and positive cells were stained brown-yellow (red arrows). Analyses of CD163-positive cells in intestine and kidney were presented in the bottom. (**E**) The concentration of TNF-α and IL-1β in intestine and kidney was detected by using ELISA assay, respectively. Analyses of TNF-α and IL-1β concentration in intestinal mucosa (upper) and kidney (lower) were presented. Data were expressed as mean±SEM, n=4 to 8. Results were compared by ANOVA with Tukey posttest. * *P* < 0.05 vs. Sham group; # *P* < 0.05 vs. MI group; $ *P* < 0.05 vs. TP group. Control group: the RAW264.7 cells were treated with DMSO; LPS group: the cells received LPS challenge; Terlipressin group: the cells received terlipressin immediately after LPS; Terlipressin+Wortmannin group: the cells received terlipressin and wortmannin in the presence of LPS. Sham group: the SMA of rat was exposed but not occluded; MI group: acute mesenteric ischemia model was produced by clamping the SMA; TP group: terlipressin was infused after unclamping the SMA; NE group: norepinephrine was infused after unclamping the SMA; WT group: wortmannin, a specific PI3K inhibitor, was used after mesenteric ischemia; T+W group: terlipressin and wortmannin were both administered after ischemia. MI: mesenteric ischemia; SMA: superior mesenteric artery; PI3K: phosphoinositide 3-kinase; TNF: tumor necrosis factor; IL: interleukin; iNOS: inducible nitric oxide synthase; hpf: high power field; ELISA: enzyme‑linked immunosorbent assay; DMSO: dimethyl sulfoxide; LPS: lipopolysaccharide.

As shown in Fig. 3A, the apoptotic index in TUNEL analysis of the intestinal epithelia was significantly higher in the MI group than in the Sham group (*P*<0.05), while the apoptosis of intestinal epithelial cells in the TP and NE groups were markedly reduced when compared with the MI group (both *P*<0.05). Moreover, cleaved capsase-3 expression notably increased in intestinal mucosal tissues after MI (*P*<0.05 vs. Sham; Fig. 4B) and was significantly reduced by the use of terlipressin or norepinephrine (both *P*<0.05 vs. MI; Fig. 3B).

The apoptotic index and cleaved caspase-3 expression in the renal tissues were also increased in the MI group (both *P*<0.05 vs. Sham; Fig. S1). However, neither terlipressin nor norepinephrine reduced renal apoptosis (all *P*>0.05 vs. MI; Fig. S1). Moreover, neither terlipressin nor norepinephrine significantly inhibited the 8-isoprostane upregulation induced by acute MI (both *P*>0.05 vs. MI; Fig. S2).

### Terlipressin inhibited macrophage M1 polarization and excessive inflammation after acute MI

As shown in Fig. 4A, *in vitro*, M1 markers (iNOS and IL-1β) mRNA expressions were significantly increased after LPS treatment (both *P*<0.05 vs. Control), whereas M2 markers (Arg1 and FIZZ1) did not significantly change (both *P*>0.05 vs. Control). Terlipressin inhibited iNOS and IL-1β mRNA expressions in RAW264.7 cells (both *P*<0.05 vs. LPS; Fig. 4A). Meanwhile, the increased TNF-α and IL-1β levels in culture medium after LPS challenge were significantly reduced by terlipressin (both *P*<0.05 vs. LPS; Fig. 4B).

**Figure 5:**
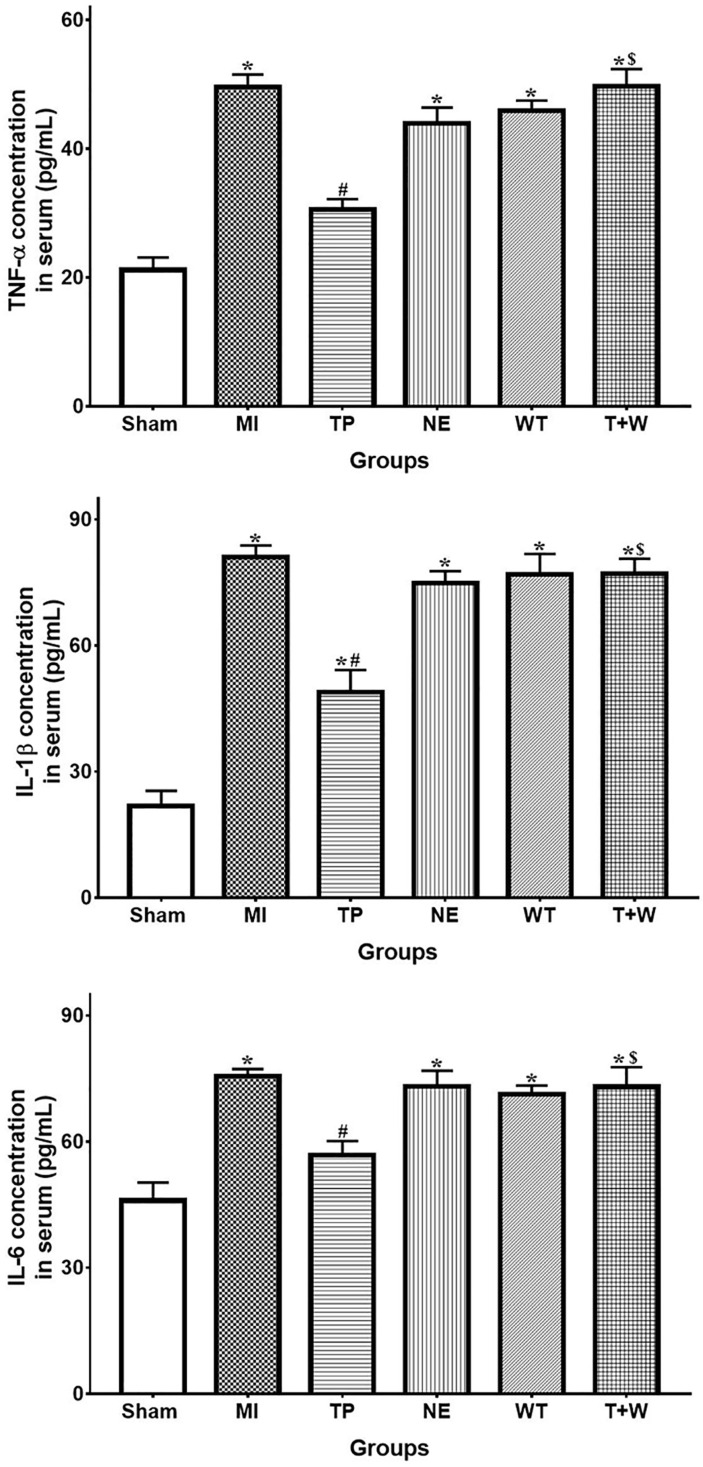
** Effects of terlipressin or norepinephrine infusion on proinflammatory cytokines in serum after acute MI.** The concentration of proinflammatory cytokines in rat's serum was detected by using ELISA assay, respectively. Analyses of TNF-α, IL-1β an IL-6 concentration in serum were presented. Data were expressed as mean±SEM, n=4 to 6. Results were compared by ANOVA with Tukey posttest. * *P* < 0.05 vs. Sham group; # *P* < 0.05 vs. MI group; $ *P* < 0.05 vs. TP group. Sham group: the SMA of rat was exposed but not occluded; MI group: acute mesenteric ischemia model was produced by clamping the SMA; TP group: terlipressin was infused after unclamping the SMA; NE group: norepinephrine was infused after unclamping the SMA; WT group: wortmannin, a specific PI3K inhibitor, was used after mesenteric ischemia; T+W group: terlipressin and wortmannin were both administered after ischemia. MI: mesenteric ischemia; SMA: superior mesenteric artery; PI3K: phosphoinositide 3-kinase; TNF: tumor necrosis factor; IL: interleukin; ELISA: enzyme‑linked immunosorbent assay.

Furthermore, the number of iNOS-positive cells were significantly increased in intestinal and renal tissues in group MI (both *P*<0.05 vs. Sham; Fig. 4C), but the number of CD163-positive cells did not significantly change (both *P*>0.05 vs. Sham; Fig. 4D). Terlipressin, but not norepinephrine, reduced the number of iNOS-positive cells after MI (both *P*<0.05 vs. MI; Fig. 4C), whereas CD163-positive cells were not significantly changed by terlipressin (both *P*>0.05 vs. MI; Fig. 4D). Similarly, the increased concentration of TNF-α and IL-1β in the intestinal and renal tissues caused by acute MI were reduced in the group TP (all P<0.05 vs. MI; Fig. 5E) but not in the group NE (all *P*>0.05 vs. MI; Fig. 4E).

In rat's serum, the concentration of TNF-α, IL-1β and IL-6 were higher in the group MI (all *P*<0.05 vs. Sham; Fig. 5). Terlipressin significantly decreased the levels of these cytokines in the serum (both *P*<0.05 vs. MI), whereas norepinephrine failed to decrease the concentration of TNF-α, IL-1β and IL-6 (all *P*>0.05 vs. MI; Fig. 5).

### Terlipressin relieved organ injuries after acute MI via PI3K/Akt pathway

**Figure 6:**
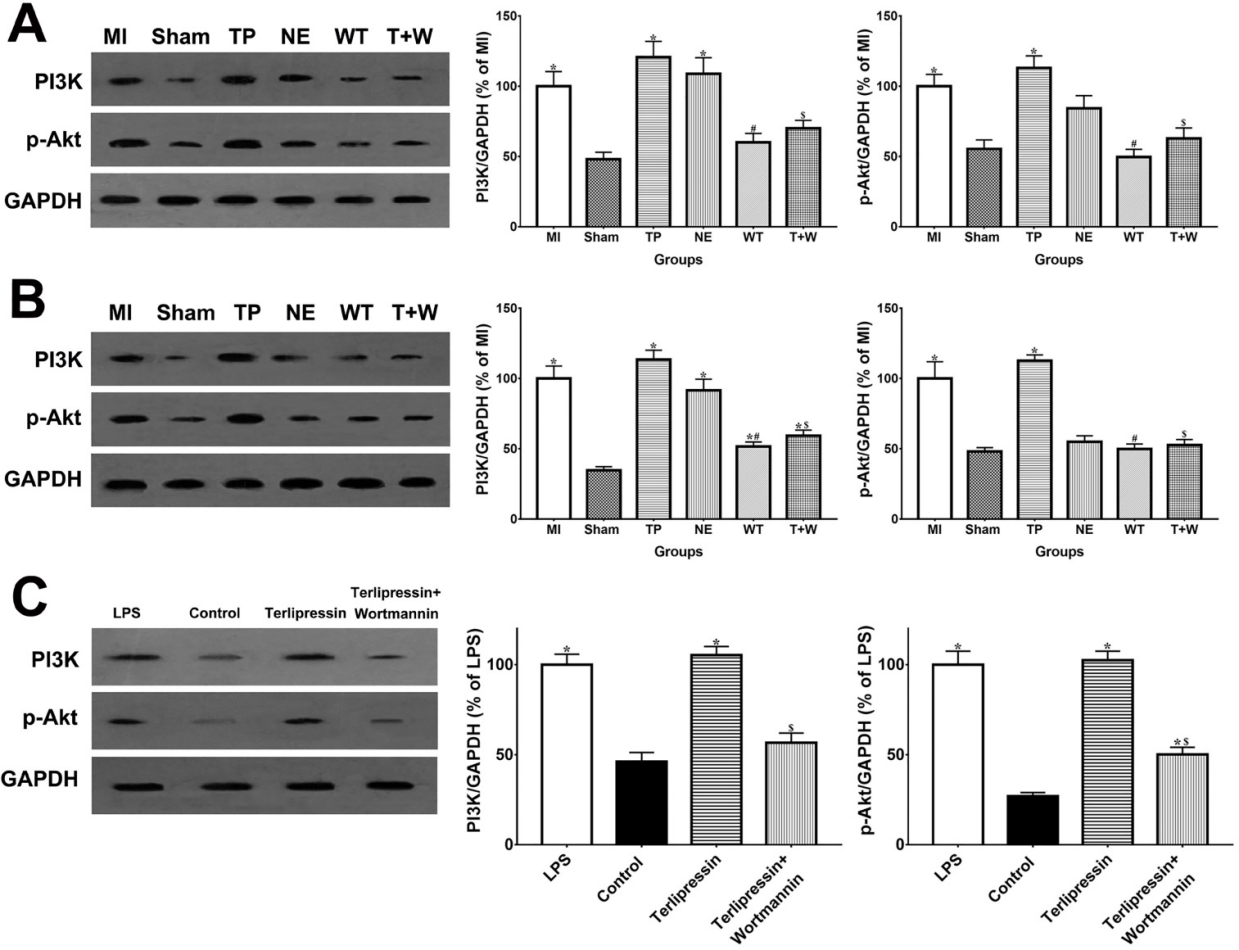
** Effects of terlipressin or norepinephrine on PI3K and p-Akt expression in cultured cells after LPS attack and in organs after acute MI.** The PI3K and p-Akt expression was detected by western blotting assay in intestinal mucosa (**A**), in kidney (**B**) and in RAW264.7 cells (**C**), respectively. Representative bands of densitometry analysis from each group were shown in the left side, and analysis of quantitative changes in PI3K/p-Akt expressions were shown in the right side. Data were expressed as mean±SEM, n=4 to 7. Results were compared by ANOVA with Tukey posttest. * *P* < 0.05 vs. Sham group; # *P* < 0.05 vs. MI group; $ *P* < 0.05 vs. TP group. Control group: the RAW264.7 cells were treated with DMSO; LPS group: the cells received LPS challenge; Terlipressin group: the cells received terlipressin immediately after LPS; Terlipressin+Wortmannin group: the cells received terlipressin and wortmannin in the presence of LPS. Sham group: the SMA of rat was exposed but not occluded; MI group: acute mesenteric ischemia model was produced by clamping the SMA; TP group: terlipressin was infused after unclamping the SMA; NE group: norepinephrine was infused after unclamping the SMA; WT group: wortmannin, a specific PI3K inhibitor, was used after mesenteric ischemia; T+W group: terlipressin and wortmannin were both administered after ischemia. MI: mesenteric ischemia; SMA: superior mesenteric artery; PI3K: phosphoinositide 3-kinase; p-Akt: phospho-protein kinase B; GAPDH: glyceraldehyde-3-phosphate dehydrogenase; DMSO: dimethyl sulfoxide; LPS: lipopolysaccharide.

As shown in Fig. 6A and 6B, *in vivo*, the PI3K and p-Akt protein expressions in the intestinal mucosa and renal tissues were elevated after acute MI (all *P*<0.05 vs. Sham). The use of terlipressin moderately, but not statistically, enhanced PI3K and p-Akt expressions in the intestinal epithelia and renal tissues (all *P*>0.05 vs. MI; Fig. 6A and 6B). Similarly, the PI3K and p-Akt expressions in the cultured RAW264.7 cells were also elevated after LPS challenge (both *P*<0.05 vs. Control; Fig. 6C). And terlipressin slightly increased PI3K and p-Akt expressions in the RAW264.7 cells with LPS treatment (both *P*>0.05 vs. LPS; Fig. 6C).

*In vivo*, wortmannin alone (WT group) produced no active effects on the changes caused by MI (all *P*>0.05 vs. MI; Fig. 1-5) but decreased the PI3K and p-Akt expressions in the cells and tissues (all *P*<0.05 vs. MI; Fig. 6). On the other hand, wortmannin totally diminished the active effects of terlipressin on the PI3K/Akt expressions (Fig. 6), the intestinal epithelial apoptosis, macrophages M1 polarization and inflammatory activation (Figs. 3-5), and the MAP, organ damages and animal survival (Fig. 1 and 2) in the T+W group, when compared with the TP group (all *P*<0.05).

## Discussion

In the current study, occluding and unclamping of SMA successfully induced vasodilatory shock and caused intestinal and renal injury (Figs. 1 and 2). Our dosing regimens of terlipressin and norepinephrine mimicked the applications of vasoactive drugs in the clinical settings, and the current infusing doses in rats are approximately equal to usual dosage of terlipressin and norepinephrine for shock treatment in humans according to a classical conversion of drug dose from animal to human 19. Several clinical evidences suggested that terlipressin, as compared with norepinephrine, did not increased digestive complications in the treatment of vasodilatory shock and hepatorenal syndrome 20-22. However, to the best of our knowledge, no study has compared the efficacy of vasopressin receptors agonist versus norepinephrine on the organ functions and outcome following acute MI. The current data showed that either terlipressin or norepinephrine application could effectively increase MAP (Fig. 1A), improve the organ damages and reduce mortality (Fig. 2) after severe MI insult in rats.

In the present study, acute MI and following reperfusion induced sustaining and severe hypotension, whose hemodynamic data were line with previous findings 15, 23. For shock treatment, it is essential to restore MAP timely to guarantee adequate tissue perfusion and oxygen delivery. The administration of either terlipressin or norepinephrine rapidly and effectively improved MAP (Fig. 1A) and subsequently relieved organ damages following acute MI insult (Fig. 2). This suggests that the timely restoration of MAP and organ perfusion pressure delivered by these two vasoactive drugs plays an important role in the intestinal and renal protection. Besides impaired organ perfusion, excessive intestinal epithelial apoptosis and inflammation, as well as macrophage M1 polarization, were also involved in the pathogenesis of MI-induced organ injury 15, 18, 24. Apoptosis is a major mode of intestinal epithelial cell death after acute MI attack, and inhibition of apoptosis can prevent the destruction of mucosal barrier in such pathophysiological events 25-27. Two previous studies have reported that terlipressin could decrease cerebral and hepatic apoptosis in ischemic conditions 28, 29. Similarly, the results of the apoptotic index and cleaved caspase-3 expression explicitly showed that terlipressin management could significantly inhibit intestinal mucosal apoptosis (Fig. 3) in intestinal ischemia. Interestingly, neither terlipressin nor norepinephrine inhibited renal apoptosis following MI (Fig. S1). Apoptosis in distant organs caused by acute MI needs further investigations.

Our previous study suggested that M1 macrophages polarization was promoted after acute MI, and that macrophage depletion or enhancing switch from proinflammatory M1 to anti-inflammatory M2 macrophages phenotypes could ameliorate intestinal injury and improved survival 18, 30. Chang and Jan found that, vasopressin inhibited the activation of cultured macrophages and the release of proinflammatory cytokines following endotoxin challenge 31, 32. To the best of our knowledge, the current study for the first time showed that, both *in vitro* and *in vivo*, V1 receptor agonist markedly blocked macrophages M1 polarization after acute MI challenge whereas it produced no distinct effect on M2 polarization (Fig. 4). We speculate that LPS migrated from the gut lumen induced macrophages M1 polarization in various organs after MI, and that increased M1 macrophages and subsequently produced excessive inflammatory responses led to tissue injury and organ failure. Because V1 receptor agonist possesses anti-inflammatory effects 31, 32, terlipressin, as compared with norepinephrine, potently restrains the activated M1 macrophages in various organs and then reduces the release of inflammatory cytokines after acute MI (Figs. 4 and 5). The present data strongly indicated that terlipressin offered reliable and efficient protections for acute MI-induced multiple organ dysfunction when compared with traditional vasopressor. These novel findings may help laboratory investigators and clinicians to deeply understand mechanism of distant organ injury caused by MI and to choose the optimal vasoactive drugs in MI-relevant diseases.

In cardiovascular system, PI3K inhibitors in particular have the potential to reduce blood pressure 33, and PI3K inhibitors could block the phenylephrine-induced arterial contraction and reduce MAP under a volatile anesthetic sevoflurane conditioning 34. Vasopressin receptors are G protein-coupled receptors and PI3K has been implicated in a signal transducer of G protein-associated signaling. It has been demonstrated that the vasoconstricting effect of vasopressin through V1 receptor is mediated by phosphotidylinositol pathway 35. Interestingly, by activating PI3K/Akt signaling, vasopressin also exerts its ability to inhibit the activation of downstream NF-κB and the upregulation of inflammatory mediators in macrophage induced by endotoxin 32. It has been widely proven that the activation of PI3K/Akt signaling could reduce the M1 polarization by downregulating the TLR signaling and activating mTOR which then decreases the NF-κB activation 36, 37. Previous studies have shown that Akt isoforms hold the key role in the regulation of macrophage activation 36, 38. Specifically, Akt1 kinase contributes to M2 polarization while Akt2 kinase contributes to M1 polarization due to their opposite roles in regulating miR-155 and its target, C/EBPb, a master regulator of macrophage differentiation 38. We speculate a dominant activation of Akt2 kinase but not Akt1 in current MI model and thus only M1 marker changed notably. Also, our previous study and Miller's study have demonstrated the anti-apoptotic effect of vasopressin and terlipressin via activating PI3K/Akt signaling 13, 39. The current data demonstrated that wortmannin could diminish the hemodynamic improvement induced by terlipressin after MI insult whereas wortmannin alone produced no active effect (Fig. 1A), suggesting that PI3K inhibitor may abolish V1 receptor-mediated vasoconstricting effect. Wortmannin also significantly eliminated the protective effects of terlipressin on reducing macrophage M1 polarization and attenuating organ damages after MI challenge by inhibiting PI3K activation (Fig. S3). Our clinically meaningful results suggest that vasopressin V1 receptor agonists are not suitable for patients with PI3K inhibitors therapy (e.g. treatment and prevention of cancer).

There were some possible limitations in this study. First, the present results indicated that terlipressin provides organ protection by elevating blood pressure and inhibiting apoptosis and M1 polarization. However, it is very difficult to distinguish whether the main effect of terlipressin on MI insult is attributed to the improved organ perfusion or the repression of apoptosis and M1 polarization. Second, in this experimental study, severe hypotension was induced by intestinal ischemia and reperfusion. Whereas, in the clinical settings, hypotensive shock in the patients with acute MI was usually induced by other critical conditions (e.g. sepsis and hemorrhage). Final, in this study, we speculated that decreased M1 macrophages generation (Fig. 4) caused by terlipressin may play a key role in the suppression of systemic inflammation (Fig. 5). Further verification is required to confirm this hypothesis.

Taken together, MI leads to vasodilatory hypotension and notable intestinal and renal injury. The use of terlipressin or norepinephrine effectively improves hemodynamics and organ damages, and subsequently reduces mortality. Terlipressin delivers its protective effects by elevating blood pressure and inhibiting intestinal epithelial apoptosis and macrophage M1 polarization via PI3K/Akt pathway. Our study exhibits that terlipressin may be a promising vasoactive alternative for MI relevant conditions in clinical practices.

## Supplementary Material

Supplementary figures and tables.

## Abbreviations

MI: Mesenteric ischemia
ICU: Intensive care unit
SMA: superior mesenteric artery
NS: normal saline
TNF-α: tumor necrosis factor-α
IL: interleukin
PI3K/Akt pathway: phosphoinositide 3-kinase/protein kinase B pathway
ELISA: Enzyme Linked Immunosorbent Assay
DAO: Diamine oxidase
BUN: blood urea nitrogen
GAPDH: glyceraldehyde-3-phosphate dehydrogenase
TUNEL: terminal deoxynucleotidyl transferase biotin-dUTP nick end-labeling assay
