# Supplementary material for: Terlipressin relieves intestinal and renal injuries induced by acute mesenteric ischemia via PI3K/Akt pathway
Source: Int J Med Sci. 2020 Sep 28;17(17):2751–62. doi: 10.7150/ijms.46302 (PMC7645354; doi:10.7150/ijms.46302)
Supplement: Supplementary file 1 — Supplementary figures and tables. [file ijmsv17p2751s1.pdf]

Fig. S1 Effects of terlipressin or norepinephrine infusion on renal apoptosis after acute MI. (A) The renal sections were stained by using TUNEL and evaluated with apoptotic epithelial cells under light microscopy (×400). Representative microscopic images from various groups were presented in the upper panel, and apoptotic nuclei were stained dark brown (red arrows). Analysis of apoptotic index was presented in the lower left-hand. (B) The cleaved caspase-3 expression in renal tissues was detected by western blotting assay. Representative bands of densitometry analysis from each group were shown in the upper side, and analysis of quantitative changes in caspase-3 expressions was shown in the bottom. Data were expressed as mean±SEM, n=4 to 7. Results were compared by ANOVA with Tukey posttest. * $P < 0.05$ vs. Sham group; # $P < 0.05$ vs. MI group; $ $P < 0.05$ vs. TP group.

Sham group: the SMA of rat was exposed but not occluded; MI group: acute mesenteric ischemia model was produced by clamping the SMA; TP group: terlipressin was infused after unclamping the SMA; NE group: norepinephrine was infused after unclamping the SMA; WT group: wortmannin, a specific PI3K inhibitor, was used after mesenteric ischemia; T+W group: terlipressin and wortmannin were both administered after ischemia.

MI: mesenteric ischemia; SMA: superior mesenteric artery; PI3K: phosphoinositide 3-kinase; TUNEL: terminal deoxynucleotidyl transferase biotin-dUTP nick end-labeling; c-caspase-3: cleaved caspase-3; GAPDH: glyceraldehyde-3-phosphate dehydrogenase

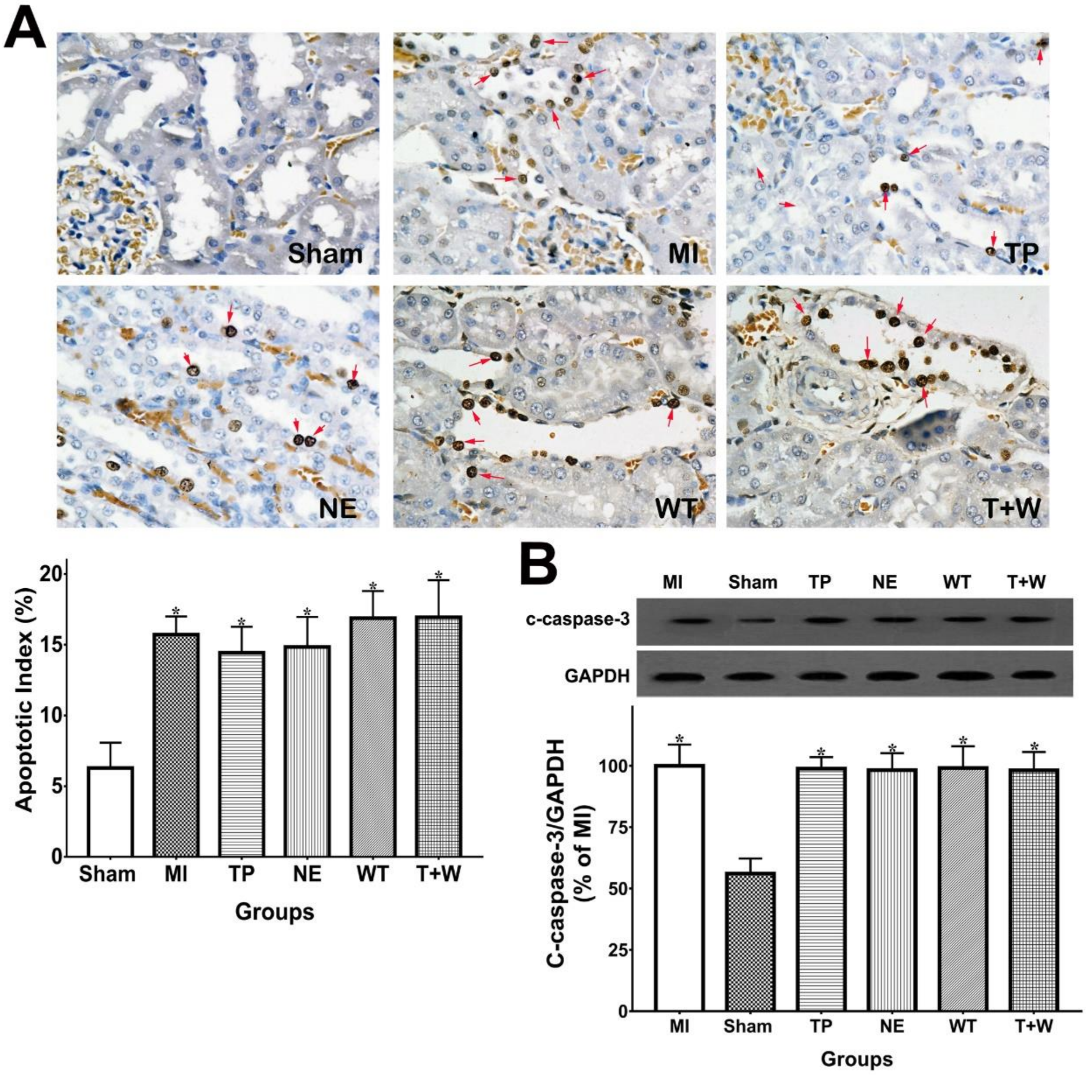
A
Sham
MI
TP
NE
WT
T+W
Apoptotic Index (%)
20
15
10
5
0
Sham
MI
TP
NE
WT
T+W
Groups
B
MI
Sham
TP
NE
WT
T+W
c-caspase-3
GAPDH
C-caspase-3/GAPDH (% of MI)
100
75
50
25
0
MI
Sham
TP
NE
WT
T+W
Groups

Fig. S2 Effects of terlipressin or norepinephrine infusion on 8-isoprostane in serum after acute MI. The concentration of 8-isoprostane in serum was detected by using ELISA assay. Analyses of 8-isoprostane concentration was presented. Data were expressed as mean±SEM, n=4 to 6. Results were compared by ANOVA with Tukey posttest. * $P < 0.05$ vs. Sham group; # $P < 0.05$ vs. MI group; \$ $P < 0.05$ vs. TP group. Sham group: the SMA of rat was exposed but not occluded; MI group: acute mesenteric ischemia model was produced by clamping the SMA; TP group: terlipressin was infused after unclamping the SMA; NE group: norepinephrine was infused after unclamping the SMA; WT group: wortmannin, a specific PI3K inhibitor, was used after mesenteric ischemia; T+W group: terlipressin and wortmannin were both administered after ischemia.

MI: mesenteric ischemia; SMA: superior mesenteric artery; PI3K: phosphoinositide 3-kinase; ELISA: enzyme-linked immunosorbent assay

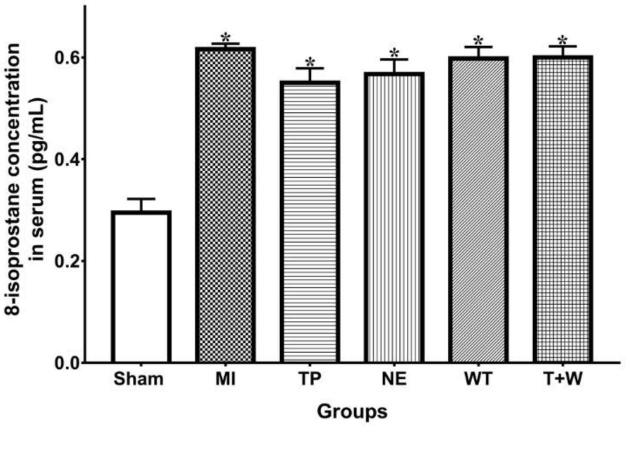

Fig. S3 The proposed Terlipressin's mechanism for relieving intestinal and renal injury caused by acute MI. In the current rat model, acute MI led to severe hypotension and cause notable intestinal impairments. The gut barrier dysfunction further promoted inflammatory response and macrophages M1 polarization in intestinal and renal tissue. Terlipressin restored the mean arterial pressure, reduced apoptosis in intestinal and renal tissue, and reduces macrophages M1 polarization of by activating the PI3K/Akt pathway. Wortamanin almost totally counteracted the above protective effect of terlipressin.

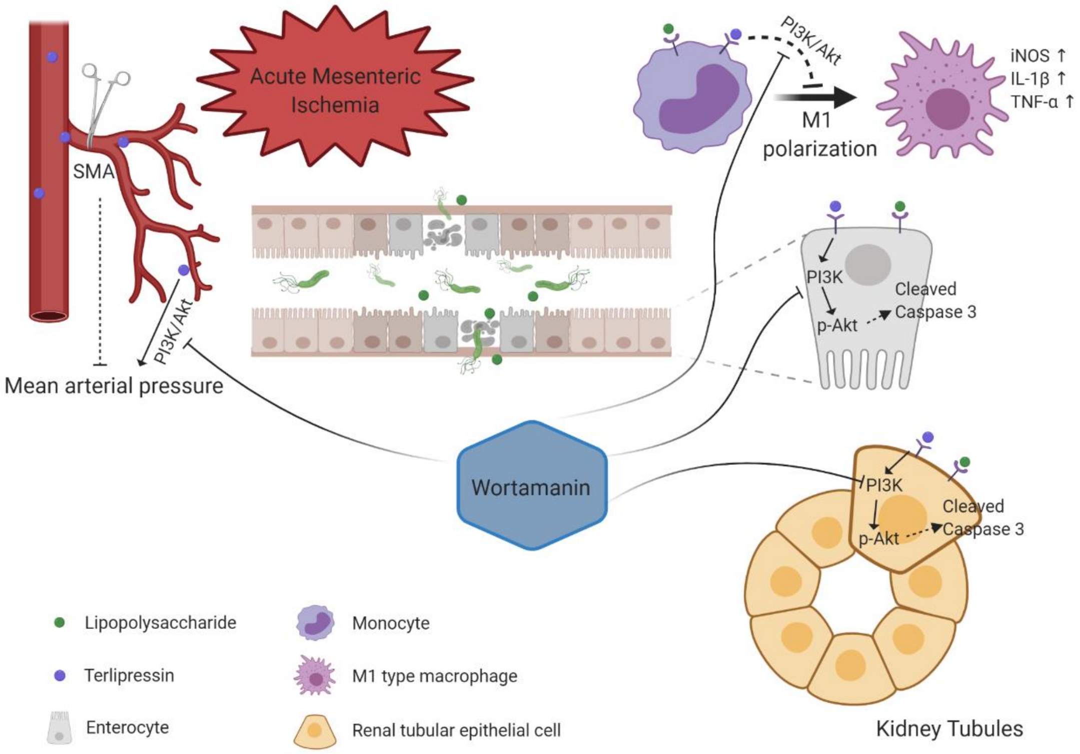

Supplementary Table

Table S1.
Damage scoring system for intestinal mucosa (Chiu's score)

Score	Histological change of mucosa
0	normal villi
1	mild development of subepithelial Gruenhagen spaces
2	moderate progressive lifing of the epithelial layer from the lamina
3	propria
4	severe progressive lifing of the epithelial layer from the lamina propria
5	completely denuded villi
	disintegration of the lamina propria

Damage scoring system for kidney histological injury
Histological change: dilation or loss of Bowman's space, flattening of renal tubular epithelium, loss of tubular brush border, microhemorrhage, and tubular casts

Score	Grade
1	<10%
2	10 to 25%
3	26 to 75%
4	>75%
